# An Aggressive Case of Mucormycosis

**DOI:** 10.7759/cureus.9610

**Published:** 2020-08-07

**Authors:** Donovan Tran, Berndt Schmit

**Affiliations:** 1 Diagnostic Radiology, University of Arizona College of Medicine - Tucson, Tucson, USA; 2 Radiology, University of Arizona College of Medicine - Tucson, Tucson, USA

**Keywords:** rhinocerebral mucormycosis, mucormycosis, rhizopus, invasive fungal sinusitis, retroantral fat, isavuconazole

## Abstract

Mucormycosis is an aggressive fungal disease that can occur in individuals with certain predisposing factors, such as diabetes mellitus and pharmacologic immunosuppression. An astounding aspect of this disease is the speed at which it can spread to surrounding structures once it begins to germinate inside the human body. This case involves a 24-year-old male patient who presented to the emergency room complaining of a headache after a dental procedure who developed fulminant rhinocerebral mucormycosis within days. The objective of this report is to shed light on how fast this disease spreads, discuss current management of rhinocerebral mucormycosis, and illustrate the subtle, but critical radiographic findings to raise clinical awareness for this life-threatening disease.

## Introduction

We share our world with fungi. They are ubiquitous in nature; current estimates put the number of fungal species to be as high as 5.1 million [1]. As plentiful as they are, only hundreds of these species are pathogenic to humans, collectively killing more than 1.6 million people annually [2]. Common fungi that cause illness are Aspergillus species, Candida albicans, Cryptococcus neoformans, Blastomyces dermatitidis, and Rhizopus species. The term mucormycosis refers to any fungal infection caused by fungi belonging to the Mucorales order [3]. Fungi usually enter the body through inhalation of spores but can also gain access through open wounds. Most healthy people effectively clear fungal elements from their bodies by the innate immune system. However, people with specific risk factors such as diabetes mellitus, immunosuppression, hematologic malignancies, renal failure, organ transplant, and high iron overload are more prone to developing fungal infections [3].

## Case presentation

A 24-year-old male patient with no known past medical history presented to the emergency department with a severe headache and right-sided facial pain. He endorsed increased thirst during the previous few days and a family history of diabetes. He denied any history of fever, chills, nausea, vomiting, or abdominal pain. On the prior day, the patient had a wisdom tooth extraction without any complications. On the day following the procedure, he began to experience severe headache and right-sided facial pain. The patient went back to the dental clinic to be checked out and was found to be hypertensive and severely hyperglycemic. He was immediately transferred to the nearest hospital for the treatment of diabetic ketoacidosis. Four days later, the situation worsened. The patient developed increased right-sided swelling of the face with proptosis and complete visual deterioration bilaterally. His left pupil was constricted while his right pupil was fixed and dilated. He had lagophthalmos, an inability to completely close his eyelids. His mental status declined and intubation was required. Subsequent imaging of the head was obtained and showed concern for an invasive fungal rhinosinusitis.

**Figure 1:**
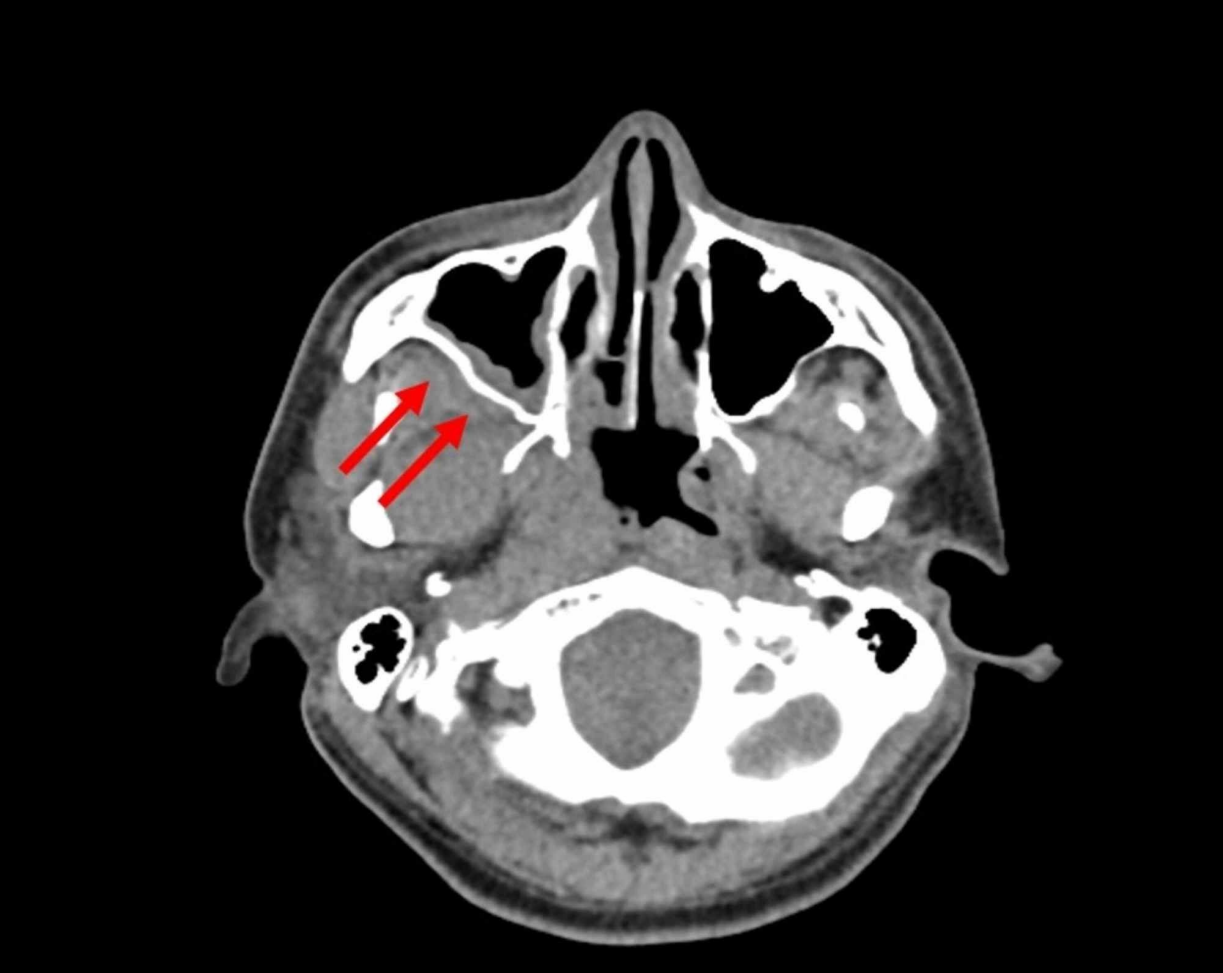
Axial non-contrast CT on admission. The red arrows show infiltration of the right retroantral fat plane indicating invasive disease extending through the posterior wall of the right maxillary sinus.

**Figure 2:**
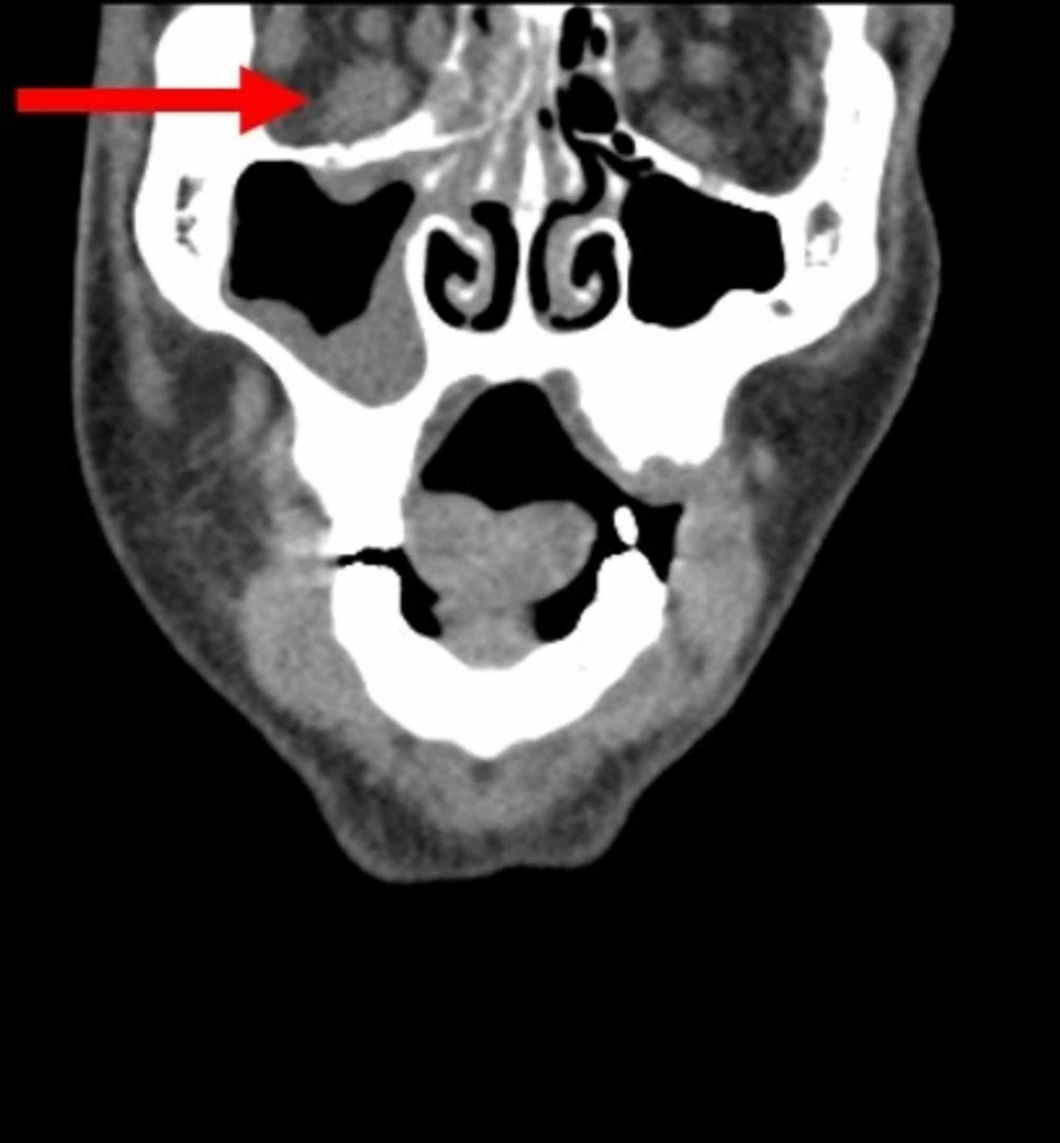
Coronal non-contrast CT on admission. The red arrow shows marked enlargement and indistinctness of the right inferior rectus muscle indicating inflammatory changes.

On initial arrival to the hospital, the patient was found to be hypertensive. He had a systolic pressure of 163 mmHg and a diastolic pressure of 110 mmHg. Labs showed a leukocyte count of 16,000/mm^3^, a blood glucose of 424 mg/dL, an HbA1C of 1.1, pH of 6.99, potassium of 2.6 mEq/L, serum bicarbonate of 6.8 mEq/L, an anion gap of 33 mEq/L, and ketonuria. A CT scan of the neck was performed that showed infiltration of the right retroantral fat plane, a subtle but critical finding (Figure 1). This radiographic finding indicates that the disease has invaded through the bone of the right maxillary sinus. Marked enlargement and indistinctness of the right inferior rectus muscle indicative of an inflammatory process were also seen on initial imaging (Figure 2). 

**Figure 3:**
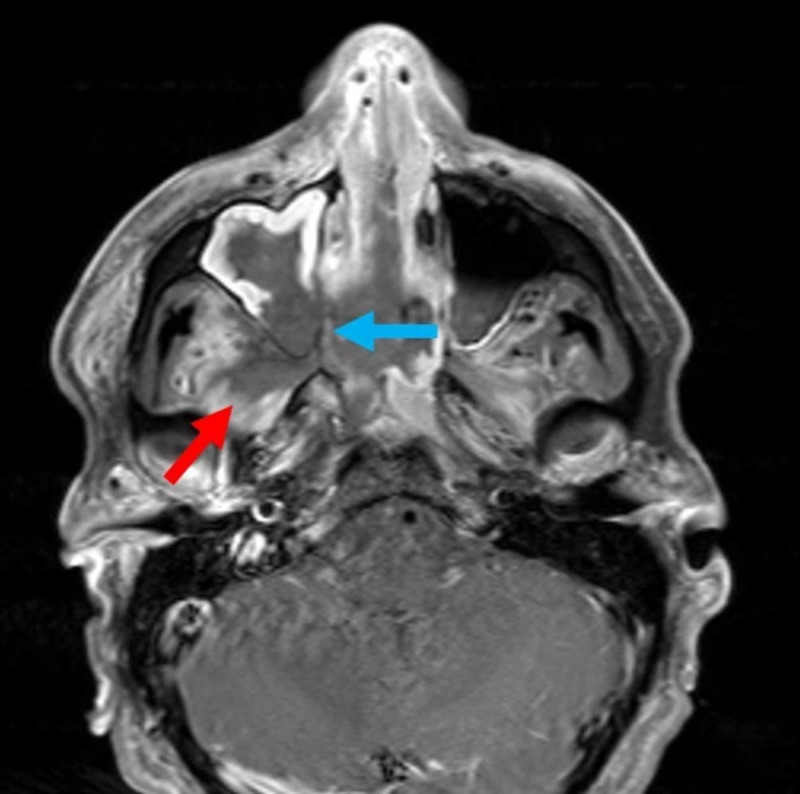
Axial T1 fat-saturated postcontrast MR image on day 2 of admission. There is marked enhancement of the bilateral pterygoid muscles indicating infection. The red arrow shows non-enhancing portion of the pterygoid muscles indicating necrosis. Worsening mucosal thickening and enhancement of both maxillary sinuses from spreading infection. The blue arrow shows non-enhancing mucosa of the right maxillary sinus indicating mucosal necrosis.

Two days later, MR scans demonstrated multifocal regions of mucosal necrosis and inflammatory disease involving the sinonasal cavity diffusely and extending into the bilateral orbits and right masticator space causing pterygoid muscle necrosis (Figure 3). 

**Figure 4:**
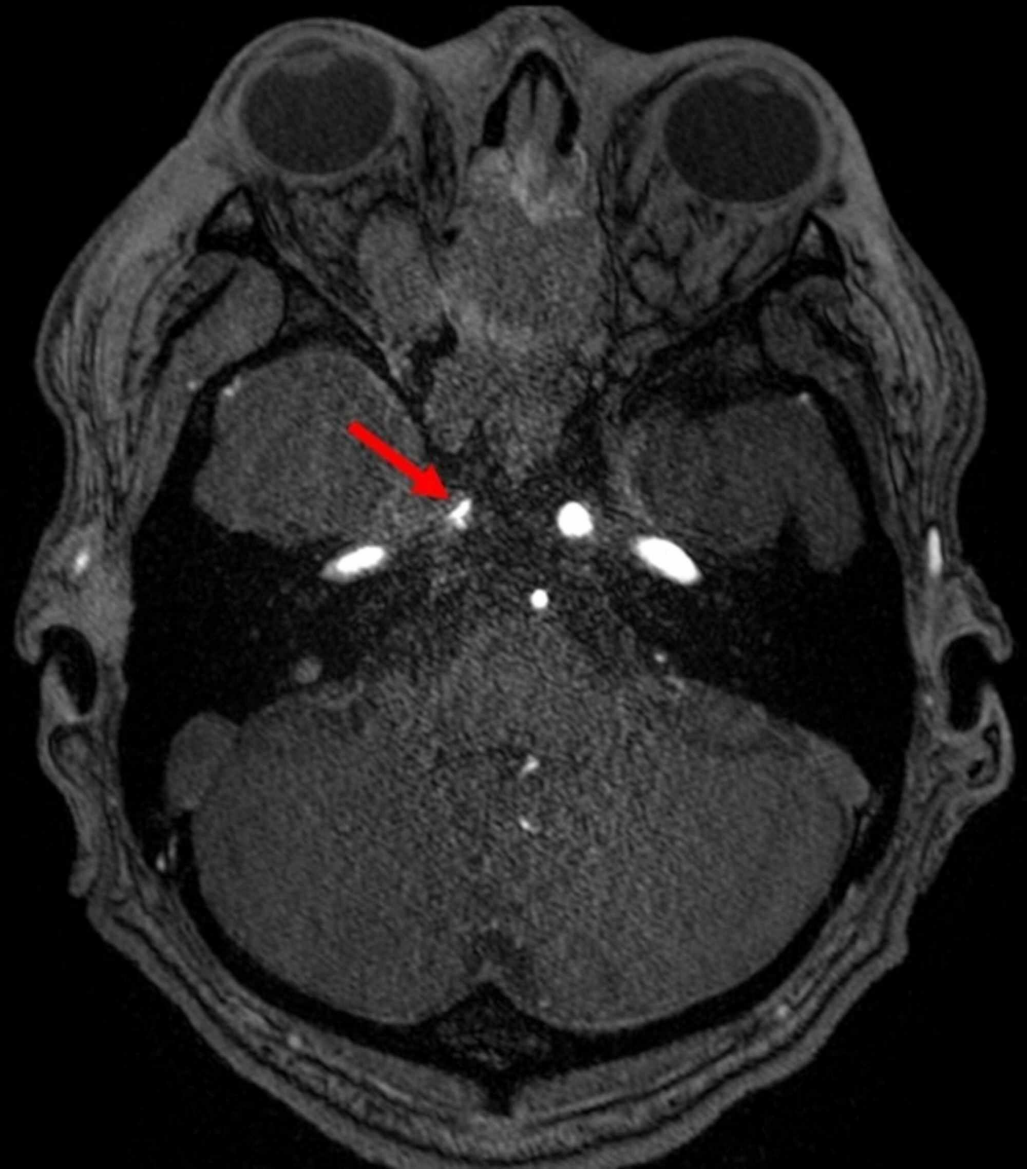
Axial time of flight MR angiography through the cavernous sinus on day 2 of admission. The red arrow shows marked irregular narrowing of the right internal carotid artery indicating direct invasion of the carotid artery.

**Figure 5:**
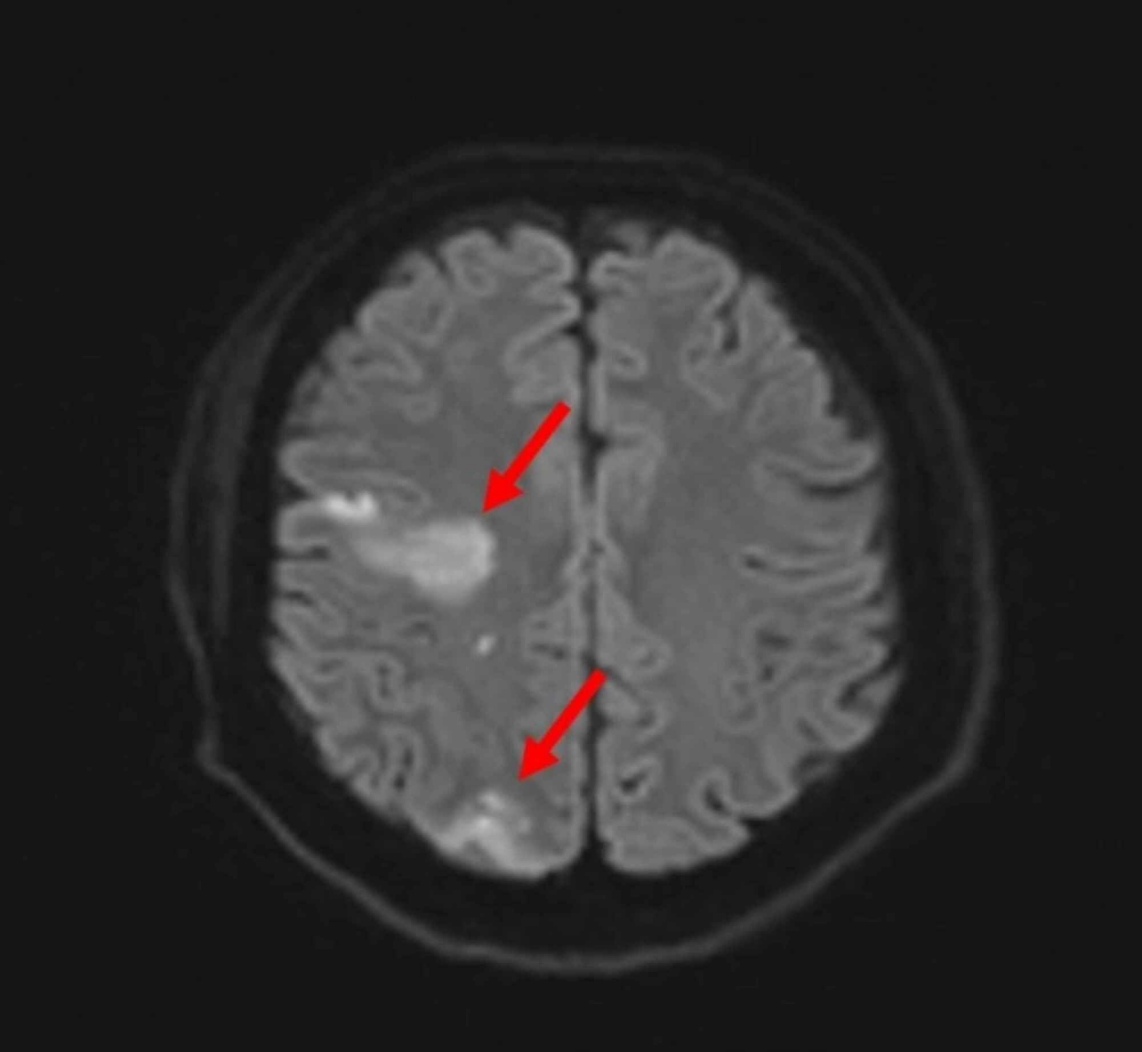
Axial MR diffusion-weighted image on day 2 of admission. The red arrows show areas of acute infarction of the right frontal and right parietal lobes.

Unfortunately, the fungus also invaded the right internal carotid artery causing arteritis and cavernous sinus thrombosis with embolic infarcts of the right frontal and parietal lobes (Figures 4, 5). Tissue cultures from the right maxillary sinus and right infratemporal fossa grew methicillin-sensitive Staphylococcus aureus, Cutibacterium, and Rhizopus species. These findings confirmed a diagnosis of invasive mucormycosis. 

Management of this patient’s invasive fungal rhinosinusitis required multidisciplinary collaboration. Diligent communication and effort among the Internal Medicine, Ophthalmology, Otolaryngology, Infectious Disease, and Neurology teams were critical to this patient’s care. The patient was given IV fluids, started on an insulin drip and liposomal amphotericin B, and transferred to an intensive care unit for management. Due to the continuous insulin drip and amphotericin B treatment, he was frequently hypokalemic and required persistent potassium replenishment. The insulin infusion was stopped if the patient’s potassium level dropped below 3.5 mmol/L to prevent hypokalemia-induced cardiac arrhythmias. Once the patient’s hyperglycemia was controlled, the insulin drip was replaced with a regiment consisting of insulin glargine, insulin lispro, and a sliding scale for strict glucose goal of 140-180 mg/dL.

He was started on broad-spectrum antibiotics: vancomycin with a trough goal of 15-20 mg/L, meropenem 1 g every eight hours, isavuconazonium 372 mg every eight hours for six doses followed by 372 mg daily, liposomal amphotericin B 5 mg/kg/day, metronidazole 500 mg every six hours, and erythromycin ophthalmic ointment. Throughout his hospital course, nafcillin and posaconazole were also given. Posaconazole was discontinued because serum levels were found to be subtherapeutic (less than 0.7 mcg/mL). The patient was also given Neupogen® due to persistent neutropenia likely due to long-term vancomycin exposure.

To contain the invasive disease, Otolaryngology conducted extensive surgical debridement that was performed three times during the hospital course. Operations included resection of the anterior skull base, septoplasty, bilateral sinus debridement, bilateral medial orbital wall decompression, right pterygopalatine and infratemporal fossa debridement, and bilateral sinus surgery with Draf III. Despite over a month of antibiotic and antifungal treatment, there was complete necrosis of the right eye and extra-ocular muscles. Due to the high risk of harboring infection, the decision to remove the right orbit was made to reduce future intracranial disease.

The patient’s initial prognosis was poor. The fungal infection had caused extensive damage to his sinonasal cavity, right pterygopalatine fossa, skull base, cavernous sinus, and multiple lobes of the brain, resulting in mucosal, vascular, and nerve injury. However, with continued antifungal treatment, his mental status slowly improved. He became hemodynamically stable, was able to end ventilatory support, answer and ask questions, and follow commands. The patient made a remarkable recovery. However, his vision never recovered in either eye. He also had persistent left proptosis, left-sided facial weakness, and right-sided facial sensory loss. The patient was discharge to a skilled nursing facility after a prolonged hospital stay of 48 days.

At a two-month follow-up outpatient appointment with Otolaryngology, he was found to have improved extra-ocular movements of his left eye and no signs of invasive fungal sinusitis. Follow-up MRI showed stable left intra-orbital phlegmon and myositis of the extra-ocular muscles and intracranial disease. He will require continued surveillance of the right cavernous sinus thrombophlebitis, right internal carotid artery arteritis, and multiple intracranial infarcts. He will possibly require life-long isavuconazonium treatment.

## Discussion

Rhizopus is an aggressive, rapidly invasive fungal species that can cause mucormycosis. Within only four days of presenting with headache and facial pain, our patient developed severe proptosis, acute loss of vision, and deteriorating mental status that required intubation.

Any fungi in the Mucorales order can lead to mucormycosis. The most common cause is Rhizopus species, with Mucor species coming in second [4]. Rhizopus species have a high moisture requirement to grow and thus are not typically found inside buildings. Rather, they tend to be found outdoors in soil and decaying material. Normally, these fungi do not cause disease in healthy people. However in people with underlying health conditions or immunosuppression, they may cause serious rhinocerebral, pulmonary, gastrointestinal, cutaneous, or disseminated infections [5].

Risk factors that predispose a patient to mucormycosis are diabetes mellitus, neutropenia, malignancy, recurrent diabetic ketoacidosis, iron overload syndromes, and corticosteroid use. It has also been found that the use of iron chelators is a risk factor [6].

The most common route of entry for fungi is through the respiratory tract. Sporangiospores travel through the nares and deposit in the nasal turbinates. From there, they can gain access to the pulmonary system. Other routes of entry include through scrapes in the skin or through ingestion of contaminated foods where they can cause cutaneous and gastrointestinal mucormycosis, respectively [5]. Our patient’s infection was apparently induced by the wisdom tooth extraction. There have been several other reports of rhinocerebral mucormycosis occurring after dental extractions [3]. It is possible that the fungal spores gain access through the open mucosal wound.

Once inside the human body, the spores swell, germinate, and progress to germ tube formation with hyphal extension. The hyphae invade the tissues and have a tendency to invade blood vessels where they cause thrombosis and tissue necrosis [7].

Most healthy humans clear mucormycosis through the innate immune system. With the help of the alternative complement pathway, phagocytic cells and neutrophils destroy the spores, preventing germination. In diabetic patients, mucormycosis cannot be controlled. There is evidence that hyperglycemia and acidosis impair the ability of phagocytes to kill the spores through their normal oxidative burst and non-oxidative mechanisms [7].

Additionally, fungi require iron for growth and virulence. Conditions leading to high serum iron levels, such as diabetic ketoacidosis (as seen in our patient) or hemochromatosis, predispose patients to mucormycosis. Acidosis likely disrupts the binding ability of iron-binding proteins, such as transferrin, allowing the fungi to utilize the free iron for growth. Certain iron chelating agents such as deferoxamine have been shown to increase the risk of mucormycosis, while others such as deferiprone and deferasirox may be toxic to fungi [6]. Deferasirox has been successfully used as salvage therapy to treat a patient with rhinocerebral mucormycosis who failed months of polyene treatment.

Rhino-orbito cerebral involvement is the primary location of infection, such as in our patient [7]. Early signs of infection include fever, headache, proptosis, facial pain, and swelling. Once the fungi establish a colony in the nasal structures, it can extend to surrounding structures. Mucormycosis can involve the paranasal sinuses and the orbit. If it extends to the retro-orbital region, cranial nerves III, IV, and VI can become damaged leading to loss of extraocular movements, dilated pupils, and vision loss. Cavernous sinus thrombus from hematogenous spread has been reported in some cases [8]. In our patient, the infection grew to involve both orbits causing multifocal regions of necrosis of the extraocular muscles and bilateral exophthalmos. Additionally, vascular inflammation led to right-sided cavernous sinus thrombophlebitis and arteritis of the right internal carotid artery.

Diagnosis of mucormycosis starts with having a high clinical suspicion of invasive fungal disease. Patients with diabetes mellitus with signs of rhinosinal disease should have immediate CT imaging. Subtle imaging findings may indicate invasive infection. Tissue sampling should also be conducted to confirm the diagnosis. Under direct microscopy, the fungi will have ribbon-like, non-septate hyphae with a diameter ranging from 5 to 25 μm and 90° branching angles. If mucormycosis is still suspected after a negative culture result, molecular identification may be performed. Different techniques such as DNA probe targeting 18S subunit and ITS1 after polymerase chain reaction with pan-fungal primers have been reported [9].

The treatment of mucormycosis requires a combination of surgical debridement and antifungal therapy. Depending on the extent of the necrosis, surgical debridement of necrotic tissue can involve removing sections of the maxilla, nasal cartilage, palate, mandible, and the orbit [8]. The mainstay antifungal therapy is amphotericin B. Our patient received IV liposomal amphotericin B at a starting dose of 5 mg/kg daily for 18 days before increasing the dose to roughly 10 mg/kg for another 18 days. Isavuconazonium 372 mg every eight hours was also given in combination with liposomal amphotericin B and tapered to one dose daily. Unlike posaconazole, isavuconazole does not require routine serum level monitoring because therapeutic levels are reached in most patients with standard dosing regiments [10].

It is crucial that empiric antifungal treatment is initiated early. Delaying amphotericin B therapy increases mortality. A retrospective cohort study involving 70 patients with hematologic malignancies with mucormycosis showed that patients receiving delayed amphotericin B (six days after symptom onset) had a twofold increase in the mortality rate at four weeks compared to patients receiving earlier treatment [11].

One of the main side effects of amphotericin B is nephrotoxicity. In our patient, creatinine levels began to slowly rise after 22 days of receiving amphotericin B. Although still within normal limits, the creatinine increased from a baseline of 0.67 to 1.62 mg/dL. It has been widely reported that amphotericin B can cause renal potassium and magnesium wasting from tubular injury; therefore, electrolyte serum levels should be frequently monitored. A pseudohyperphosphatemia may also occur with administration of amphotericin B. This is likely due to interference of amphotericin B with the phosphate assay in the laboratory [12]. Liposomal amphotericin B has been shown to have fewer nephrotoxic effects compared to conventional amphotericin B.

Obtaining control of blood glucose levels with insulin treatment is also critical to containing the infection. In our patient, a glucose goal of 140-180 mg/dL was targeted. Care must be taken to make sure the serum potassium levels do not drop to dangerous levels which can predispose the patient to cardiac arrhythmias. Ensuring closure of the anion gap and restoration of electrolyte abnormalities were important to this patient’s recovery. With the combination of extensive surgical debridement and aggressive antifungal therapy, the fungal infection was ultimately controlled, but unfortunately for this patient, he will nevertheless suffer long-term significant sequelae. 

## Conclusions

Mucormycosis is an aggressive fungal infection that arises particularly in patients with medical conditions such as hyperglycemia, acidosis, or iron overload (i.e. diabetes mellitus, hemochromatosis, iron chelator use). It is crucial that empiric treatment with amphotericin B and/or isavuconazonium should be initiated early to decrease mortality. The aim of this report is to bring clinical awareness to the subtle radiographic findings that may help diagnose this aggressive entity. Clinicians must maintain a high index of suspicion for mucormycosis.
